# The Role of Social Media in Making an Impact to Health Knowledge and Behaviour on COVID-19 in Malaysia

**DOI:** 10.21315/mjms2021.28.3.15

**Published:** 2021-06-30

**Authors:** Aneesa Abdul Rashid, Irfan Mohamad, Ahmad Firdaus Mohd Haris

**Affiliations:** 1 Department of Family Medicine, Faculty of Medicine and Health Sciences, Universiti Putra Malaysia, Serdang, Selangor, Malaysia; 2 Islamic Medical Association of Malaysia (IMAM) Response and Relief Team (IMARET), Cheras, Kuala Lumpur, Malaysia; 3 Department of Otorhinolaryngology-Head and Neck Surgery, School of Medical Sciences, Universiti Sains Malaysia, Kubang Kerian, Kelantan, Malaysia; 4 Persatuan Pembanteras Mitos Perubatan Malaysia (Medical Mythbusters Malaysia), Batu Caves, Selangor, Malaysia; 5 Jabatan Kesihatan Negeri Perak, Ipoh, Perak, Malaysia

Dear Editor,

We read with great interest the article entitled ‘The behaviour changes in response to COVID-19 pandemic within Malaysia’ published by the *Malaysian Journal of Medical Sciences* (MJMS) in April 2020 (1). The authors discussed the essential topic of behaviour response amidst the global coronavirus disease 2019 (COVID-19) pandemic and how it affected behaviour. Although some behaviours are similar to those of past disasters, it is undeniable that these behaviours are highly influenced by social media.

In reference to this, we would like to highlight how social media has become an essential component of the general public and a powerful communication medium, especially those that involve and affect a global audience. The world’s response to COVID-19 was a social media storm, with everyone having an opinion to share (2). A study conducted between February and March 2020 regarding COVID-19 topics in Twitter reported more than 160,000 unique tweets. These tweets mainly revolved around the knowledge of COVID-19 and its impact (3). Locally, the average reach for an article or post on the Medical Mythbusters Malaysia’s Facebook page, an NGO dedicated to sharing accurate health information on social media, was 53,574 Facebook reach per article. The highest COVID-19 themed post for May was a reach of 194,600 unique Facebook users (4). Facebook reach is defined as the number of unique users who referred to particular content, thus illustrating the high demand for social media information.

Considering the era of social media, this appears to be the new norm. However, the kind of information being shared and the individuals sharing them is a serious concern. Recent reports have shown worrying trends on the kind of advice shared through this medium. Authors of a YouTube video content analysis conducted in March 2020 reported that more than 25% of the most viewed videos on COVID-19 contain misleading information (5). These types of information disseminate faster and may potentially influence the community’s reaction to uncertain topics. This highly relates to the point the authors of the article had emphasised upon — ‘groupthink’ and ‘conformity’ — which is highly influential in the realm of social media. As a result, inaccurate information that quickly disseminates may cause unnecessary panic and anxiety to the community. For example, in South Africa, researchers had evaluated 11 cases of confirmed xenophobic fake-news-related-content between 2015 and 2019, and although social media may not be solely responsible, they found mounting evidence suggesting that it acted as a vehicle to spread tensions between the locals and the migrant population that subsequently lead to an escalation of racial tensions (6).

The notion that social media caters more towards the ‘younger generation’ and that it is not a credible source of information needs to change for the better. Another perturbing fact is that the academic and medical communities are not in line with this reality, which could be attributed to the fact that some find it less significant while others find the concept of expert communication via social media alienating. A study conducted on health researchers and clinicians on social media reported that only 15% of them use social media for professional reasons, such as sharing research knowledge, while more than half believe they needed more training on how to use it (7). This, therefore, leads to the increased influence of false experts or pseudo-experts on social media, amplifying our concern on the dissemination of misleading information highlighted above. However, medicine has rapidly advanced over the last 20 years and it is gradually in-sync with the digital age (8). Medical professionals have to swim at a faster pace in this mainstream knowledge sharing business; otherwise, medical myths will continue to dominate the belief amongst the ‘netizens.’

Nevertheless, there are certain steps taken in Malaysia, specifically in terms of effective communication sharing on social media, which is commendable. Keeping the social media world updated via official channels has helped the public come to terms with embracing proper knowledge and information. An example is the setting up of an official website by the Ministry of Health Malaysia to disseminate proper guidelines of the COVID-19 (9). Not to mention, the undeniable support of the Malaysian community towards fundraising efforts of non-governmental organisations (NGO) mainly steered by social media. IMARET is one of the many NGOs that have teamed up with social media influencers, raising more than RM2 million in funds to help the COVID-19 crisis, while sharing proper and timely information on the pandemic (10, 11). IMARET launched its COVID-19 funds on the 17th of March concentrating efforts on assisting front liners, besides providing essential medical equipment and supplies. In addition, the NGO assisted marginalised, B40 communities and families affected by the quarantine. IMARET now plans to reach out and assist neighbouring countries. It also uses social media platforms to disseminate reliable information, garnering more than 20,000 followers combined.

We reiterate here that much more proactive efforts in terms of research and health policies are needed to address this issue. It is essential to acknowledge that social media is an important platform to communicate health information to the public.
